# 

**Published:** 1900-12

**Authors:** 


***NOTICE.—This JOURNAL and THE INTERNATIONAL JOURNAL OF SURGERY one year for $1.00 cash, strictly to new subscribers. No out-of-town checks accepted unless 10c. is added for cost of cashing. Strictly to new subscribers.***

THE JOURNAL-RECORD desires to congratulate Dr. George H. Noble upon the collapse of the suit against him. Those who are familiar with Dr. Noble's unusual dexterity as an operator and his uniformly careful, painstaking and successful surgical work will doubly applaud the justice of the verdict and his complete vindication.

**This Journal and Atlanta Semi-weekly Journal one year for $1.00 cash in advance. No checks. Strictly to new subscribers.**

